# Opportunities for CMS to improve healthcare access and equity through advancing technology-enabled startups and digital health innovations

**DOI:** 10.1038/s41746-024-00997-x

**Published:** 2024-01-30

**Authors:** Shobha Dasari, Raihana Mehreen, Kristin Baker Spohn, Andrey Ostrovsky

**Affiliations:** 1 Department of Computer Science, Stanford University, Stanford, CA USA; 2 Research Manager, Social Innovation Ventures, Rockville, MD USA; 3 Lecturer, Stanford Graduate School of Business, Stanford, CA USA; 4 Managing Partner, Social Innovation Ventures, Rockville, MD USA

**Keywords:** Health policy, Health services

## Abstract

Historically, the Centers for Medicare and Medicaid Services (CMS) has formed partnerships with select private sector entities, including large traditional hospital and health system networks, nursing homes, and payer groups. However, innovations from technology-enabled services companies and digital technology companies are uniquely poised to aid CMS in addressing key barriers toward advancing its mission of improving healthcare access and equity. There are four pivotal opportunity areas where partnerships with technology businesses and tools would enhance the work of CMS: (1) improving consumer awareness about CMS programs, (2) mitigating access gaps through virtual care programs, (3) streamlining the complexity of different payer plan models, and (4) using technology-enabled services to address social risk factors without imposing additional burdens on providers. We offer examples of digital and technology-enabled solutions that improve patient access to care and close equity gaps, as well as propose specific recommendations for CMS to advance and expand the reach and impact of these solutions. Namely, these recommendations include partnerships with private sector companies that can educate and support consumers about their benefits, the extension of telehealth reimbursement parity for virtual care solutions, allowing for cross-state licensure across plans and reimbursement for care coordination services that alleviate provider burden to screen and address patients’ social determinants of health needs. We argue that CMS has an imperative role in leveraging the innovations of technology-enabled services and digital health technologies to lower healthcare access barriers, mitigate provider burden, stimulate innovation, and close equity gaps at the patient, provider, and innovator levels.

## Introduction

The Centers for Medicare and Medicaid Services (CMS) has made concerted efforts over the last few years to improve health access and equity. Many of these efforts attempt to address disparities in health and healthcare in the United States, especially among racial and ethnic minorities, individuals dually eligible for Medicare and Medicaid, and those living in rural and underserved areas, who are more likely to face challenges with accessing healthcare services, lower quality of care and below-average health outcomes when compared to the general population^1^.

Historically, CMS’s partnerships with private sectors have included larger organizations and traditional healthcare providers, including hospital and health system networks, nursing homes, and payer groups (such as through Medicare Advantage and Managed Medicaid)^2^. We believe that innovations from small businesses in the private sector and technology-enabled startups are uniquely poised to aid CMS in addressing key barriers toward advancing its mission of improving healthcare access and equity. Our insights were formed with the partnership of 15 digital health and health equity experts, who have identified pivotal challenges pertaining to burdensome policies and complexities at the patient, provider, and innovator levels.

The expert discussion yielded three major themes. First, consumers generally lack awareness of their eligibility for CMS programs and face barriers in navigating these programs. We recommend increased and centralized education and outreach initiatives to improve consumer awareness. Second, there is a strong opportunity to improve access to care through virtual solutions. Third, the complexity between different payer plan models impedes provider availability, creates unnecessary burdens, and contributes to provider burnout. We recommend standardizing credentialing and billing infrastructure and operations among plans, as well as implementing cross-state licensing to alleviate provider shortages. Lastly, we recommend that CMS invests in methods to identify and determine the impact of social risk factors without increasing provider burden, as the effects of increased medical and social complexity disproportionately fall on providers without commensurate compensation or support.

There is a pressing need for CMS to leverage the innovations of technology-enabled startups and small businesses to mitigate the barriers described above. In the subsequent sections, we highlight the potential for small businesses and digital technologies to alleviate healthcare access barriers and reduce complexities for patients and providers, as well as to spur innovation, improve healthcare access and experience and advance health equity.

## Close access gaps by enhancing awareness of CMS benefits and programs

Consumers lack awareness in regard to eligibility for CMS programs and face obstacles in navigating them. There are a multitude of factors that contribute to these impediments, including difficulty accessing government information about benefits, lack of access to a regular primary care provider, lack of provider awareness of benefits offerings, and a fragmented patient referral process to access services. These factors prevent patients from being aware of the benefits and treatments that they are eligible for through their health plan and taking advantage of them.

Information about benefits is often complex, with most benefits having specific eligibility criteria and requirements, separate application processes, and variations by state. Rarely do states provide a centralized repository of all benefits offered, with benefits being published across legislation, agency guidance, bulletins, websites, and social media. The complexity in the communication of benefits information creates unnecessary barriers for patients in understanding which benefits they are eligible for and taking action towards receiving benefits.

In a recent survey of caregivers across the United States, 33% indicated that their top barrier was a lack of awareness of existing benefits offerings due to a lack of information, and 29% indicated difficulty in enrolling in programs or knowing their eligibility for benefits. Additionally, the process by which to apply for benefits, which often utilizes separate paperwork for each benefit, can be time-consuming for caregivers. Startup technology solutions allow caregivers to access tax credits, state programs, health plan reimbursements, and other benefits that they may be entitled to in one centralized location^3^. These platforms are able to proactively recommend benefits that caregivers may not have known about, verify caregivers’ eligibility for benefits, and offer a single, streamlined enrollment process for all benefits through a user-friendly web application. CMS can further help consumers access programs and benefits by enabling the reimbursement of benefits navigators, centralized education and referral networks, digitized summaries of benefits to enable consumers to search for available benefits, and other tech-enabled front-door solutions.

Additionally, the unwinding of the Public Health Emergency (PHE) has caused over 3.8 million individuals to lose Medicaid coverage, as states will no longer receive enhanced federal funding to keep Medicaid beneficiaries covered through the duration of the PHE^4,5^. Many individuals who have lost coverage were not notified that they were losing eligibility, with HHS estimating that 7.9% (6.8 million) will lose Medicaid coverage despite retaining eligibility^6^. Digital health platforms are able to streamline enrollment and re-certification for Medicare members in all state and federal programs that they qualify for, unlocking thousands of dollars in savings for some of the most vulnerable Americans^7^. Partnerships with such organizations will enable CMS to communicate with individuals about their benefits eligibility, reduce fluctuating health insurance churn, and improve the utilization of health benefits. CMS may also partner with groups such as Google, which recently launched improvements to its search engine to support users who search for information about re-enrolling in Medicaid, as this is a traditionally complex and opaque process. By leveraging the technological capabilities of companies like Google, CMS may improve consumer awareness about Medicaid redeterminations, increase the usability of their Medicaid enrollment processes, and disseminate accurate information about Medicaid re-enrollment in different states^8^.

## Mitigating access gaps through telehealth and virtual care solutions

In addition to this lack of awareness, patients face significant barriers in accessing care due to long waitlists and travel times, provider shortages, and limited opening hours for many healthcare facilities, which prevent beneficiaries with jobs or other responsibilities from scheduling or attending appointments. Patients in the U.S. spend, on average, two hours in wait and travel time for a 20-min visit, and this burden is 25–28% longer for racial/ethnic minorities and unemployed individuals, who typically seek health services at community health centers^9^. For specialty services, such as cardiac rehabilitation or substance abuse treatment, patients remain on the waiting list for several months, resulting in member dropouts and poorer outcomes^10^. Even patients with Medicare Advantage transportation benefits struggle to obtain care due to the ultimate lack of transportation.

Fortunately, there are virtual resolutions that offer increased access to health care for patients, especially given the social complexities of those covered under Medicare and Medicaid. During the COVID-19 pandemic, CMS allowed for the coverage of designated Category 3 Telehealth services (services that likely have a clinical benefit but lack sufficient evidence to justify permanent coverage) through the end of CY 2023^11^. Maintaining telehealth reimbursement parity flexibility to offer coverage and reimbursement for Category 3 telehealth services will allow innovations such as telehealth alcohol treatment programs and virtual care providers for dementia to enhance access to care, especially in rural and underserved areas, where patients need to travel for hours or days to the nearest center for these services (one study of Medicaid and Medicare patients in North Carolina showed that 1 in 3 patients experienced transportation barriers to access healthcare services)^12–14^. Solutions such as virtual cardiac rehabilitation and text-based, trauma-informed mental health care encompass innovative models that offer access to high-value health provisions for those who would otherwise be unable to acquire this care due to transportation and cost barriers^15,16^. Through initiatives to increase access to and coverage of virtual services among health plans, CMS has a key opportunity to close health equity and healthcare access gaps for patients.

## Alleviate provider burden through streamlined operations for credentialing, billing, and cross-state licensing across plans

CMS has made advances in launching numerous value-based payment and service delivery models in recent years^17^. However, credentialing (the process by which providers apply for inclusion into health plan networks) and billing operations vary greatly among different states, payers, and health plans, and the administrative burden impedes provider availability and contributes to provider burnout. These inefficiencies also contribute to high billing and insurance-related costs, which range from $20 for a primary care visit to $215 for an inpatient surgical procedure, representing 3–25% of professional revenue^18^.

As an example, one expert we consulted noted that under managed care plans for complex care patients, providers needed to log into four different portals in their daily workflows (patient care, documentation, and billing) due to a lack of operational standardization among different payers, and even within different plans from the same payer. Such challenges create particularly outsized burdens on smaller practices in rural and underserved communities, which have limited resources, funding, and staff to manage the varying operations and technological infrastructures among multiple plans. As a result, there is a need for CMS to set standards for credentialing and licensing among states and plans to reduce the burden for providers.

Additionally, many areas in the U.S. suffer from a chronic clinician shortage, especially in rural and underserved areas. In 2022, the Health Resources and Services Administration estimated that 98 million people live in primary care Health Professional Shortage Areas (HPSAs), 70 million people live in dental HPSAs, and 150 million people live in mental health HPSAs^19^. Providers in these areas are especially strained, and the lack of cross-state licensures creates barriers for providers to meet capacity demands where they exist. Telehealth solutions, as well as the expansion of cross-state licensures, will alleviate this shortage in rural and underserved communities.

Providers and health systems who want to offer care coordination services (i.e., organizing and managing a patient’s care across multiple providers) to their most vulnerable patients currently struggle to manage the operational overhead of hiring and credentialing their own staff to assist their patients with complex social needs, especially across different payer plans. Specifically, providers need to comply with ever-evolving state licensing requirements, complete different and repetitive credentialing applications for every payer and plan, and wait for months to hear back to finalize their license and credentials.

By collaborating with technology-enabled virtual care coordination services, especially within primary care, CMS can enable underserved communities to access high-quality virtual and community-based care while alleviating the credentialing and staffing burden for health systems and clinics, especially smaller organizations^20^. Technology-enabled platforms allow providers to connect their patients to a virtual network of allied health professionals, such as community health workers and behavioral health specialists, who are already credentialed and trained to offer care coordination services to high-need patients. This allows clinics to offer high-touch care to their patients while minimizing the time and effort required to hire more staff members or re-train their existing staff, especially in areas with shortages of healthcare personnel. The remote nature of these services also allows staff who are physically located in one part of the country, given appropriate cross-state licensures as needed, to support patients in areas with a shortage of health care providers, alleviating the problem of medical deserts.

Notably, MediCal and the CalAIM programs have supported such programs through the reimbursement of medical, behavioral, and social services for vulnerable patients, including the unhoused^21^. This additional reimbursement, whether through additional CPT codes or increased reimbursements for Z codes, for the time that cares coordinators spend with patients enables clinics to hire additional staff or contract with outsourced providers to offer these services without adding additional burden to their existing staff. By encouraging funding or reimbursement for care coordination services in all states, CMS can enable increased clinical and social support for vulnerable patients while reducing the administrative burden on providers due to staffing shortages and complex credentialing and billing operations.

## Invest in methods to determine and address social risk factors with minimal additional provider burden

CMS released a Framework for Health Equity for 2022–2032, highlighting a focus on building the capacity of the healthcare workforce to reduce health disparities^22^. A growing body of evidence portrays that social support services can improve health outcomes and reduce costs, and members of healthcare teams have a unique role in identifying unmet social needs that lead to health disparities and connecting individuals to resources that can address those needs^23^.

As healthcare providers take on the majority of the burden of screening patients for social determinants of health (SDoH) needs without commensurate compensation or support, we recommend that CMS invest in methods to identify and determine the impact of social risk factors without increasing provider burden. A recent survey of providers at a large medical school found that 58% of providers agreed or strongly agreed that the potential benefits of collecting SDoH data outweighed the costs—however, providers overwhelmingly identified a gap between SDoH data collection and addressing patient needs and the most common concerns included the lack of infrastructure to address health-related social needs and the lack of adequate knowledge to use this information effectively^24^.

CMS can play a powerful role in providing resources for healthcare professionals to execute SDoH needs screenings while enabling providers to connect patients to resources that will meet these needs. However, conducting detailed SDoH screenings for patients, as well as researching and connecting them to relevant resources in the community, can be time-consuming for already busy providers and staff. As a result, providers often forego screening patients, much less connecting them to appropriate resources, in favor of spending their limited time on services that are billable and/or top-of-license. Enabling providers to receive reimbursement for additional time and effort spent on care coordination pertaining to addressing SDoH needs will reduce provider burden around implementing SDoH programs. This reimbursement provides an incentive for providers to dedicate more time to addressing SDoH challenges, allowing them to connect patients to vital resources effectively without adding additional financial burden to their practice or time burden to their staff.

Additionally, the ability to outsource patients with more complex needs to specialized care coordination services is an innovative approach that further alleviates the burden on healthcare providers. By collaborating with specialized services or technology-enabled remote workforces, healthcare providers can focus on their primary roles, confident that their patients are receiving comprehensive care coordination. This approach not only eases the provider’s workload but also ensures patients receive high-quality and specialized support to address their unique SDoH challenges.

Solutions may leverage the technology-enabled remote workforce of community health workers to engage struggling patients and help them address SDoH barriers, activate healthy behaviors, and improve clinical outcomes^25^. One specific platform of this nature, which uses 1-on-1 calls with mentors who engage with patients through phone calls to guide them in achieving their health goals, has shown through a non-randomized study that patients with uncontrolled diabetes achieve a 1.7-point average reduction in HbA1c levels, where each 1-point reduction in HbA1c reduces health care complications by 40% and costs by an estimated $99 per patient per month^26^. This evidence highlights the potential for technology-enabled solutions and specialized care coordination services to produce tangible improvements in clinical outcomes, reduce healthcare costs, and minimize the burden on providers. It underscores the effectiveness of such approaches in addressing complex patient needs and improving healthcare access and equity.

Additionally, to better identify and engage patients with the highest SDoH needs while minimizing additional provider burden, we espouse the utilization of additional government data sources, such as supplemental nutrition assistance program (SNAP) participant data and income data. Such information can provide valuable insights into the socio-economic factors affecting patients and enable CMS to target interventions effectively. By integrating government data sources, CMS can refine its approach to patient identification and engagement, ensuring that resources are directed where they are most needed. This not only enhances the effectiveness of care coordination but also diminishes the administrative workload on providers who would have needed to amass and analyze this information themselves.

Furthermore, health plans require more incentives to address health-related social needs for their beneficiaries and facilitate the uptake of non-medical benefits. In Medicare Advantage plans (representing 34% of Medicare beneficiaries), only 20.8% of Medicare Advantage enrollees are in a plan that offers a new supplemental benefit^27^. This limited adoption of CMS’s expansion of supplemental benefits may be due to health plans’ risk aversion and a lack of evidence on the return on investment from providing these services. By including health-improving social services (such as care coordination and benefits navigation) into the medical loss ratio and annual rate calculations and enabling Medicare fiscal intermediaries to educate patients about non-medical benefits, CMS can improve health plans’ uptake of health-related social benefits, promoting the advancement of equitable and value-based care.

## Conclusion

We discuss four main themes in order to improve healthcare access, reduce health disparities, and advance CMS initiatives. First, we recommend improved communication mechanisms through digital platforms to increase consumer awareness of benefits programs. Second, we recommend the usage of virtual care solutions in order to address access gaps, especially regarding costs and transportation. We also recommend standardizing credentialing and billing, as well as allowing for cross-state licensure across plans. Lastly, we recommend tools to decrease the provider burden of screening for and addressing patients’ social risk factors.

We recognize that the digital divide, including factors like patient digital literacy and broadband access, may be a potential obstacle to these tools in achieving health equity and care access. However, through increased reimbursement to cover technology costs and additional patient education on the side of digital health companies, these technology-enabled tools still have the potential to positively benefit patients by alleviating cost and transportation barriers to care.

Through initiatives to extend the impact of technology startup companies to streamline patient experience and reduce administrative complexity, CMS can mitigate barriers to healthcare access, stimulating innovation and closing equity gaps at the patient, provider, and innovator levels.

## Data Availability

Data sharing is not applicable to this article as no data sets were generated or analyzed during this study.
